# Radiologists’ memory as a data protection risk: a worst-case stress test for chest radiograph re-identification

**DOI:** 10.1186/s13244-026-02336-y

**Published:** 2026-06-16

**Authors:** Matthias A. Fink, Christian Römer, Matthis Ebel, Eric Frodl, Daniel Pinto dos Santos, Hans-Ulrich Kauczor, Andreas Bucher

**Affiliations:** 1 Clinic for Diagnostic and Interventional Radiology, University Hospital Heidelberg, Heidelberg, Germany; 2 Translational Lung Research Center Heidelberg, Member of the German Center for Lung Research, Heidelberg, Germany; 3 Clinic for Radiology, University Hospital of Münster, Münster, Germany; 4 Institute for Mathematics and Computer Science, University of Greifswald, Greifswald, Germany; 5 Department of Diagnostic and Interventional Radiology, Goethe University Hospital Frankfurt, Frankfurt am Main, Germany; 6 Department of Radiology, University Medical Center Mainz, Mainz, Germany

**Keywords:** Confidentiality, Data anonymization, Radiography, thoracic, Memory, Artificial intelligence

## Abstract

**Objectives:**

To estimate an upper bound of memory-based re-identification risk for chest radiographs by testing radiologists under conditions that favor recognition.

**Materials and methods:**

In this prospective, multicenter, web-based reader study, radiologists from 38 centers completed two reading phases. In Phase 1, each reader interpreted ten chest radiographs. After a minimum interval of 24 h, Phase 2 included six follow-up target examinations and six new non-target examinations (50% target prevalence). After each Phase-2 examination, readers indicated whether they remembered the patient. Following a positive response, they were asked separately whether they remembered the Phase-1 pseudonym and/or case position. Thirty-three readers with fully classifiable Phase-2 data contributed 396 Phase-2 examinations to the predefined primary analysis.

**Results:**

Readers answered “remember” in 139 of 396 examinations (35.1%). Sensitivity for repeated target examinations was 50.0% (99/198), whereas 20.2% of new non-target examinations were nevertheless judged as remembered (40/198). Explicit identifiers were attempted in 23 of 396 examinations (5.8%). At least one explicit identifier, defined as the Phase-1 pseudonym and/or case position, was correct in five of 396 examinations (1.3%). In a low-prevalence model with one known patient per dataset, the positive predictive value of a “remember” response decreased from 2.44% in datasets of 100 radiographs to 0.25% in datasets of 1000 radiographs.

**Conclusions:**

Even in a design that favored memory, correct recall of explicit identifiers was rare, whereas false-positive recognition remained common. These findings support treating radiologists’ memory as a limited, upper-bound component of re-identification risk, rather than assuming that familiarity routinely translates into identification.

**Critical relevance statement:**

Even under conditions deliberately favoring memory, radiologists rarely converted familiarity with prior chest radiographs into correct explicit identifiers; in low-prevalence datasets, false-positive recognition dominated the practical meaning of a “remember” judgment.

**Key Points:**

- How often does a radiologist’s feeling of familiarity with a previously seen chest radiograph translate into correct recall of a usable explicit identifier?
- In a deliberately memory-favoring design, sensitivity was 50.0%, but 20.2% of new examinations were false positives and only 1.3% yielded a correct explicit identifier.

**Graphical Abstract:**

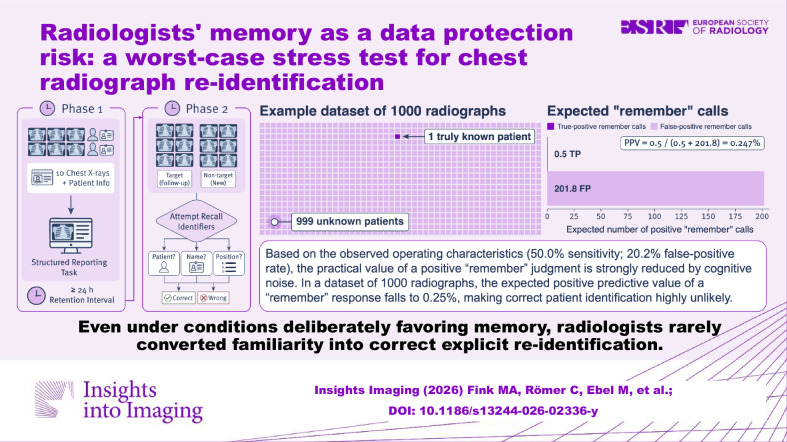

## Introduction

The secondary use of radiological images is part of the routine research infrastructure. Chest radiographs and other imaging studies are reused for educational purposes, clinical research, and developing artificial intelligence (AI) tools [1, 2]. While these activities are scientifically valuable, they depend on governance frameworks that define when image data can be shared and the degree of de-identification proportionate to the residual privacy risk [3–6]. In practice, these decisions impact whether departments can compile teaching files, assemble research cohorts, and contribute images to multicenter repositories.

Technical de-identification is necessary but not always sufficient. Patient information may still be present in DICOM headers, in overlaid text, or in the viewing environment surrounding the image [3, 7, 8]. Another concern frequently raised in discussions with ethics committees and data protection officers is that radiologists may recognize patients from memory when reviewing de-identified images in research or teaching settings [9, 10]. This issue differs from deliberate linkage attacks, data leaks, and biometric reconstruction from facial anatomy [4, 11]. It is incidental, non-adversarial, and human. Nevertheless, it can influence governance decisions and lead to highly restrictive data-sharing policies [4, 5, 12].

The available evidence for this concern is limited. Cognitive psychology shows that visual long-term memory can be strong; however, memory performance depends on image distinctiveness, task demands, and the interval between exposure and testing [13–16]. In radiology, prior studies suggest that previously viewed radiographs can be recognized at times, but the evidence base remains small. This base does not answer the most relevant question for privacy governance: Does a feeling of familiarity actually lead to the correct identification of a patient or case label [9, 10, 17]? This distinction is important because a radiologist who merely thinks an image looks familiar does not necessarily have identifying information that can be used. It also matters because the prevalence of truly known patients for any individual reader is usually very low in large research datasets.

For this reason, the present study was not designed to mimic routine clinical practice. Rather, it was designed to estimate an upper bound of risk. Two protocol features favored memory: a minimum interval of 24 h between the first and second exposures and a Phase 2 dataset in which half of the examinations had been seen before. These features facilitate recognition more than would be possible in most research repositories, where exposure intervals are often much longer and the proportion of previously encountered patients is much lower. If re-identification remains uncommon under these enriched conditions, the expected risk in routine, low-prevalence datasets should be even lower.

The purpose of this study was to quantify radiologists’ recognition of previously seen chest radiographs under controlled worst-case conditions. More importantly, the study aimed to measure how often recognition resulted in the correct recall of an explicit identifier. The goal was to provide empirical data to support proportionate governance decisions regarding the secondary use of radiographs in research, education, and AI development.

## Methods

### Study design and setting

The Ethics Committee of the Medical Faculty of Goethe University Frankfurt reviewed this prospective, multicenter, web-based reader study and confirmed that no formal ethics committee consultation was required. The committee approved the study for a waiver (reference number 2026-2823) because it used only de-identified, publicly available radiographs and allowed for anonymous participation without patient contact or new image acquisition. Conducted between February and October 2025, the study adhered to the Declaration of Helsinki and applicable data protection regulations. All participating radiologists provided electronic informed consent.

The study was performed within the German Radiological Cooperative Network (RACOON), part of the German National Research Network of University Medicine (Netzwerk Universitätsmedizin, NUM), which includes 38 university hospitals in Germany. The protocol was designed to estimate the upper bound of memory-based re-identification risk, rather than the frequency in routine clinical practice. Two design choices were made beforehand to favor memory: a minimum interval of 24 h between study phases and a Phase 2 test set comprising 50% previously seen examinations.

Throughout this manuscript, “memory-based recognition” refers to the binary Phase 2 judgment that a case was previously seen. “Explicit re-identification” refers to the correct recall of at least one Phase 1 identifier following a positive recognition judgment. The identifiers displayed within the platform were fictitious pseudonyms used solely for the experiment and were not linked to real patients.

### Reader recruitment and blinding

Readers were invited via a standardized email describing the project as a study of user interaction and structured reporting in an online thoracic radiograph reporting tool. The invitation asked participants to complete two short reading sessions on separate days. It did not mention a memory task or the re-identification hypothesis. Readers, therefore, entered Phase 1 without advance knowledge that memory would later be tested. This design choice was made to prevent artificial memorization strategies [10]. An automated reminder email was sent 24 h after Phase 1 was completed to encourage participation in Phase 2. All readings were performed independently, and readers had no access to other readers’ responses.

### Participants

Eligible participants included board-certified radiologists and radiology residents working at academic or non-academic hospitals within the network. Of the 82 registered readers, 76 completed both reading phases and were included in baseline summaries. The predefined primary analysis required a fully classifiable Phase 2 dataset across all 12 Phase 2 examinations. Thirty-three readers met this criterion and contributed 396 Phase 2 examinations. The remaining 43 completed readers had available Phase 2 responses but did not contribute a fully classifiable examination-level dataset for the predefined target/non-target performance analysis.

### Image dataset and case selection

Chest radiographs were sampled from the CheXpert dataset, a large de-identified collection of chest radiographs from clinical practice [18]. Only frontal radiographs were used. Examinations were drawn from a pool of 27 unique patients across all readers.

For Phase 1, ten different patients were randomly selected for each reader. In Phase 2, six of the initial ten patients were presented again using follow-up radiographs from the same patients (targets), together with six radiographs from previously unseen patients (non-targets). This created 12 Phase 2 examinations per reader with a target prevalence of 50%. This prevalence is much higher than in typical research datasets and was chosen deliberately to maximize the opportunity for recognition. Each examination was paired with a short clinical vignette describing a common reason for chest radiography, such as a persistent cough, shortness of breath, trauma, suspected pneumonia, or heart failure.

### Web-based reading platform and experimental procedure

**Fig. 1:**
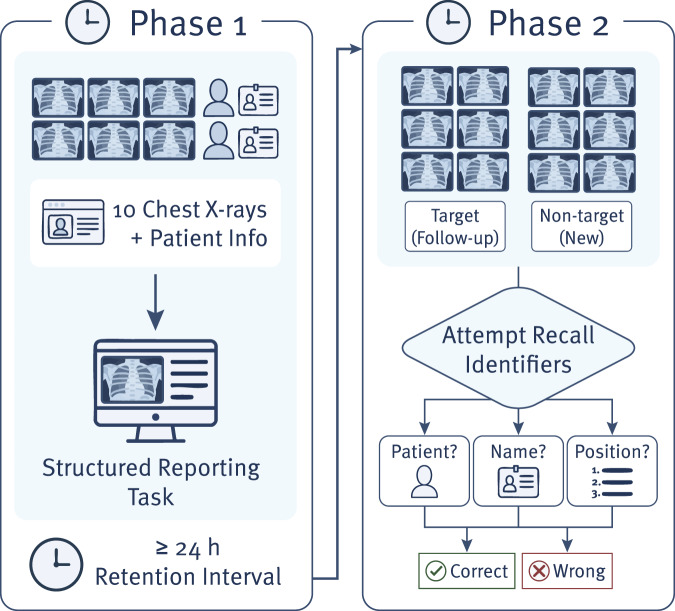
Study design and deliberate worst-case stress-test setup. In Phase 1, readers interpreted ten chest radiographs with a structured reporting task. Phase 2 became available after a minimum interval of 24 h and contained six target follow-up examinations from Phase 1 and six new non-target examinations. In Phase 2, readers indicated whether they remembered the patient and could then optionally attempt recall of the Phase 1 pseudonym and/or case position. The short interval and 50% target prevalence were chosen to favor memory and estimate an upper bound of risk

**Fig. 2:**
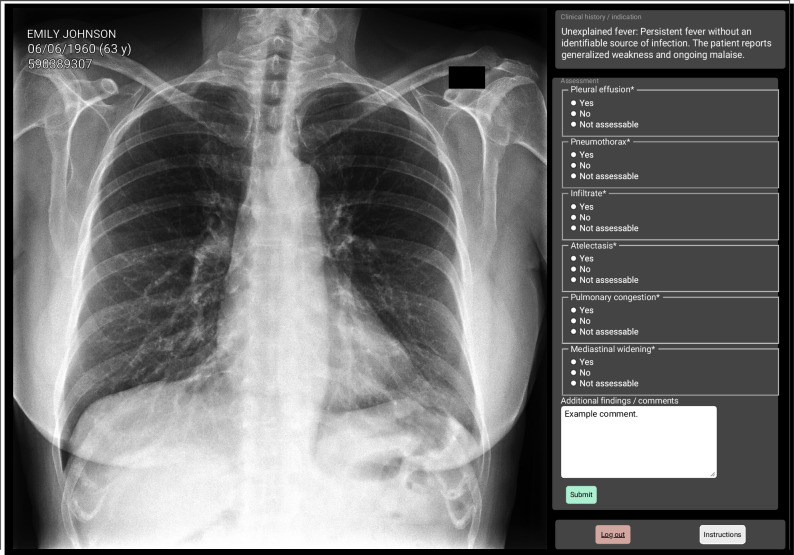
Web-based reading interface used in both study phases. Each examination was presented as a frontal chest radiograph with embedded patient pseudonym, age, and clinical indication (left) alongside a structured reporting form with predefined response options and a free-text comment field (right). In Phase 1, readers completed the diagnostic form. In Phase 2, readers answered a recall question and, after a positive response, could provide the patient pseudonym and case position in dedicated input fields. Displayed identifiers are fictitious and shown for illustration only

All readings were performed on a custom web-based platform developed for this study. Figure 1 summarizes the study design, and Fig. 2 shows an example of the reading environment. No formal training session was provided. Readers received written instructions on the screen before each phase.

### Baseline reporting (Phase 1)

In Phase 1, readers interpreted ten chest radiographs. Each examination was accompanied by a clinical vignette and a simulated patient label containing a pseudonym and either the date of birth or age, in order to emulate a routine reporting environment. Readers completed a structured reporting form containing binary items regarding pleural effusion, pneumothorax, infiltrate, atelectasis, pulmonary congestion, and mediastinal widening. There was also an optional free-text comment field. This task was intended to ensure active engagement with each examination. Readers were not informed that their memory for previously seen patients would later be tested.

### Recognition and identifier recall (Phase 2)

Phase 2 became available no earlier than 24 h after Phase 1 had been completed. Readers were shown 12 chest radiographs in random order and were asked the memory question: “Do you remember this patient from Phase 1?” After a positive response, the platform asked separately whether the reader remembered the Phase 1 pseudonym and whether the reader remembered the Phase 1 case position. Only when the reader answered “yes” to a follow-up item could the corresponding identifier be selected from a drop-down menu. Readers remained blinded to the number of repeated patients in Phase 2 and had no access to their Phase 1 reports. The platform automatically recorded the actual elapsed time between completion of Phase 1 and the start of Phase 2.

### Outcome measures

The primary endpoint for privacy interpretation was correct recall of at least one explicit identifier after a positive Phase 2 recognition judgment. Explicit identifiers were the Phase 1 pseudonym and the Phase 1 case position; therefore, correct explicit recall was defined as correct recall of the pseudonym and/or the case position. We also summarized the binary recognition judgment because familiarity without identifier recall does not identify a patient on its own.

In the primary analysis, Phase 2 examinations were classified as TP, FN, FP, or TN according to the recognition judgment. Sensitivity, specificity, overall accuracy, and predictive values of the binary recognition decision were calculated from these counts. We also described how often readers attempted explicit identifiers and how often these attempts were successful. As a secondary analysis, we report a descriptive summary of available Phase 2 responses from completed readers not included in the predefined primary analysis.

### Statistical analysis

Statistical analyses were performed by one author (M.A.F.) using R (version 4.5.1) and Python (version 3.13). Since this was an exploratory reader study without predefined effect size assumptions, no formal sample size calculation was performed. Reader characteristics are reported as the mean with standard deviation or the median with interquartile range (IQR), as appropriate. Examination-level sensitivity, specificity, accuracy, and predictive values were calculated from the aggregated confusion matrix. Examination-level 95% confidence intervals (CIs) were obtained by cluster bootstrap with resampling at the reader level. Reader-level performance is summarized as median with IQR.

Exploratory associations between performance and reader characteristics are reported descriptively. As a supplementary benchmark, examination-level sensitivity, specificity, and accuracy were compared with a random-guessing baseline derived from the overall proportion of “remember” responses using binomial tests. To illustrate the effect of prevalence on interpreting a positive recognition judgment, we modeled hypothetical de-identified datasets containing one truly known patient and *N* − 1 non-target patients for a given reader while holding the examination-level sensitivity and false-positive rate constant. The one-known-patient scenario was chosen as a transparent low-prevalence case rather than as an estimate of a specific clinical repository.

## Results

### Reader characteristics

A total of 82 radiologists registered for the study, and 76 of them completed both reading phases. Of these, 33 had fully classifiable Phase 2 data for all 12 examinations and formed the predefined primary analysis cohort. Available Phase 2 responses from the remaining 43 completed readers are summarized descriptively in Appendix E1. Table 1 shows baseline reader characteristics for all readers and for the primary analysis cohort. In the primary cohort, the median total study time was 20.3 min (IQR, 13.3–47.2 min). The interval between completion of Phase 1 and the start of Phase 2 had a median of 34.9 h (IQR, 24.6–93.1 h; range, 24.0–644.1 h).

**Table 1:** Baseline characteristics of completed readers and the primary analysis cohort

Parameter	All completed readers (*n* = 76)	Primary analysis cohort (*n* = 33)
Sex		
Male	46 (60.5%)	23 (69.7%)
Female	26 (34.2%)	8 (24.2%)
Not reported	4 (5.3%)	2 (6.1%)
Experience in radiology (years)	4.2 ± 5.4	4.2 ± 6
Reading time (min)		
Phase 1	13 (8.1, 24.4)	14.5 (8.4, 37.5)
Phase 2	3.1 (2.1, 5.5)	3.1 (2.4, 4.9)
Total reading time	19.3 (11.9, 36.8)	20.3 (13.3, 47.2)
Time between phases (hours)*	37.6 (24.5, 69.5)	34.9 (24.6, 93.1)

Unless otherwise indicated, data are frequencies with percentages in parentheses. Experience is reported as mean ± standard deviation. Reading times and time between phases are reported as medians with interquartile ranges

* Elapsed time from completion of Phase 1 to start of Phase 2

### Recognition performance

The primary analysis cohort consisted of 33 readers who contributed 396 Phase 2 examinations, including 198 target and 198 non-target cases. Readers provided a positive “remember” response in 139 of 396 examinations (35.1%). Among target examinations, 99 of 198 were judged as remembered, corresponding to a sensitivity of 50.0% (95% CI: 42.2%, 60.6%). Among non-target examinations, 40 of 198 were identified as remembered, corresponding to a false-positive rate of 20.2% and a specificity of 79.8% (95% CI: 69.6%, 87.6%). Overall examination-level accuracy was 64.9% (95% CI: 61.4, 70.6). In the study sample, which contained equal numbers of target and non-target examinations, the positive predictive value was 71.2% (95% CI: 65.1%, 79.1%) and the negative predictive value was 61.5% (95% CI: 58.9%, 66.5%). Reader-level performance metrics are detailed in Table 2. Performance exceeded the random-guessing benchmark based on the observed overall frequency of positive “remember” responses (Appendix E2).

**Table 2:** Examination-level and reader-level recognition performance in Phase 2

Parameter	Examination-level	Reader-level
Phase 2 examinations (*n*, total)	396	12
Target examinations (*n*)	198	6
Non-target examinations (*n*)	198	6
Examinations with “remember” answer	139/396 (35.1)	33.3 (25, 41.7)
Targets with “remember” answer	99/198 (50)	50 (33.3, 66.7)
Non-targets with “remember” answer	40/198 (20.2)	16.7 (0, 16.7)
TP/FN (target patients)	99/99	NA
FP/TN (non-target patients)	40/158	NA
Sensitivity	50 (42.2, 60.6)	50 (33.3, 66.7)
Specificity	79.8 (69.6, 87.6)	83.3 (83.3, 100)
Positive predictive value	71.2 (65.1, 79.1)	75 (57.9, 100)
Negative predictive value	61.5 (58.9, 66.5)	61.2 (53.4, 71.4)
Accuracy	64.9 (61.4, 70.6)	58.3 (50, 75)

Examination-level performance metrics are percentages with 95% confidence intervals in parentheses; reader-level values are medians with interquartile ranges in parentheses

*TP* true positives, *FN* false negatives, *FP* false positives, *TN* true negatives

### Explicit identifier recall

**Fig. 3:**
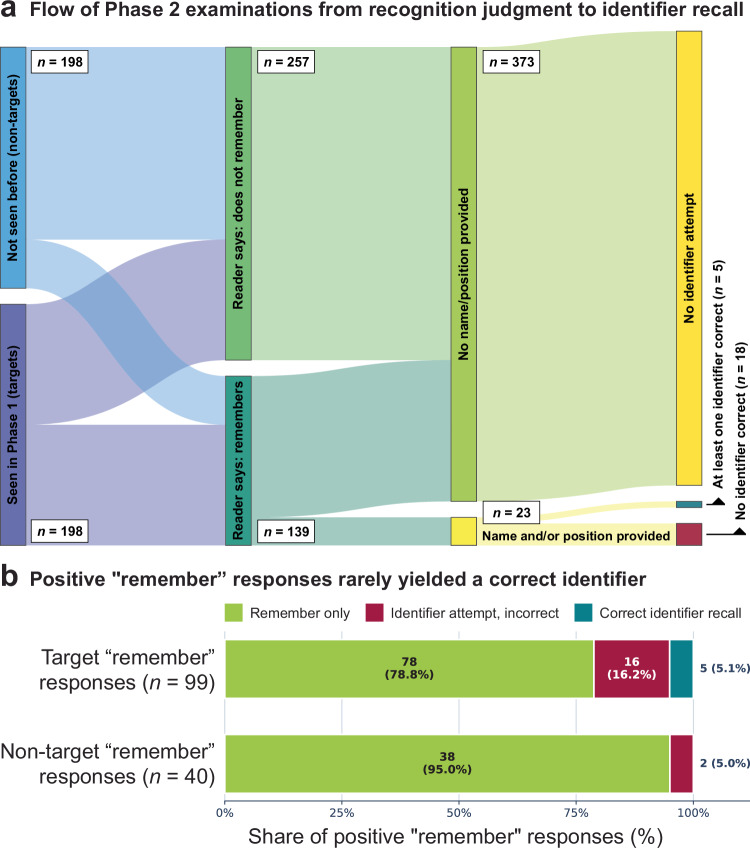
Recognition responses and conversion to explicit identifier recall in Phase 2. **a** Sankey diagram summarizing all 396 Phase 2 examinations from the 33 readers in the primary analysis cohort. Flows show whether an examination was a target or non-target, whether the reader answered “remember” or “do not remember,” whether any explicit identifier was attempted, and whether at least one identifier was recalled correctly. Flow widths are proportional to the number of examinations. **b** Decomposition of positive “remember” responses for target examinations (*n* = 99) and non-target examinations (*n* = 40) into remember-only responses, identifier attempts without correct recall, and identifier attempts with correct explicit identifier recall. Correct explicit recall was defined as correct recall of the Phase 1 pseudonym and/or the Phase 1 case position

Attempts at explicit identifier recall were made in 23 of the 396 Phase 2 examinations (5.8%) (Table 3). At least one explicit identifier was correct in five of 396 examinations (1.3%). Of the 139 positive “remember” responses, five (3.6%) yielded at least one correct explicit identifier. This corresponded to 5 of 99 target examinations with a positive recognition judgment (5.1%). Among the 99 target examinations with a positive recognition judgment, 78 (78.8%) were “remember only” responses without any identifier attempt. Three responses (3.0%) included only a pseudonym attempt, and 18 responses (18.2%) included only a case-position attempt. Two of the three pseudonym attempts were correct, whereas three of the 18 case-position attempts were correct. Among the 40 non-target examinations judged as remembered, 38 (95.0%) were “remember only,” and none of the identifier attempts were correct. Figure 3a summarizes the full flow of Phase 2 examinations from target status to recognition judgment and subsequent identifier use, and Fig. 3b decomposes positive “remember” responses into “remember only” responses, identifier attempts without correct recall, and correct explicit identifier recall. After a positive recognition judgment, pseudonym recall and case-position recall were queried separately. Among the 99 target examinations with a positive recognition judgment, readers indicated that they did not remember the Phase 1 pseudonym in 96 cases and did not remember the Phase 1 case position in 81 cases (Appendix E3).

**Table 3:** Positive recognition, identifier attempts, and correct explicit identifier recall in Phase 2

Parameter	All Phase 2 examinations (*n* = 396)	Target Phase 2 examinations (*n* = 198)	Non-target Phase 2 examinations (*n* = 198)
Recognition level			
Positive recognition (“remember”)	139/396 (35.1)	99/198 (50)	40/198 (20.2)
Any identifier attempt*	23/396 (5.8)	21/198 (10.6)	2/198 (1)
Any identifier attempt among positive recognition judgments	23/139 (16.5)	21/99 (21.2)	2/40 (5)
At least one correct explicit identifier^†^	5/396 (1.3)	5/198 (2.5)	0/198 (0)
At least one correct explicit identifier among positive recognition judgments	5/139 (3.6)	5/99 (5.1)	0/40 (0)
Pattern among positive recognition judgments			
Remember only (no identifier attempt)	116/139 (83.5)	78/99 (78.8)	38/40 (95)
Pseudonym attempt only	4/139 (2.9)	3/99 (3)	1/40 (2.5)
Position attempt only	19/139 (13.7)	18/99 (18.2)	1/40 (2.5)
Pseudonym and position attempt	0/139 (0)	0/99 (0)	0/40 (0)
Accuracy among attempts			
Correct pseudonym recall	2/4 (50)	2/3 (66.7)	0/1 (0)
Correct position recall	3/19 (15.8)	3/18 (16.7)	0/1 (0)

Values are numerators and denominators with percentages in parentheses. Rows in the “Recognition level” section use all Phase 2 examinations in the respective column as denominator unless otherwise stated. Rows in the “Pattern among positive recognition judgments” section use positive recognition judgments as denominator. Rows in the “Accuracy among attempts” section use the corresponding identifier attempts as denominator

* Any identifier attempt was defined as an attempt to recall the Phase 1 pseudonym and/or the Phase 1 case position after a positive recognition judgment

^†^ Correct explicit identifier recall was predefined as correct recall of at least one explicit identifier, that is, the Phase 1 pseudonym and/or the Phase 1 case position

### Secondary descriptive results

Among the 43 completed readers not included in the predefined primary analysis, 127 of 516 available Phase 2 examinations (24.6%) received a positive “remember” judgment, whereas identifier attempts remained uncommon (17 of 516, 3.3%; 17 of 127, 13.4% among positive “remember” judgments) (Appendix E1). Because a fully classifiable examination-level target/non-target dataset was not available for this cohort within the predefined primary analysis framework, sensitivity, specificity, and correctness-based performance measures were not estimated for these data.

### Extrapolation to low-prevalence datasets

**Fig. 4:**
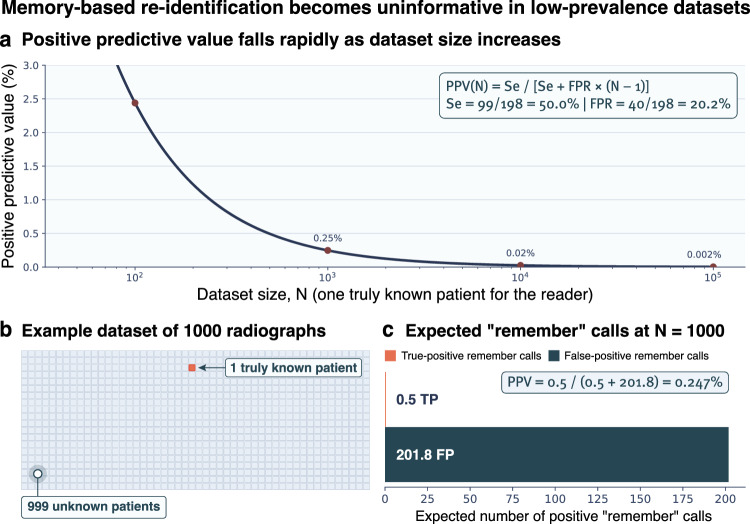
Memory-based re-identification has low practical value in low-prevalence datasets. **a** Expected positive predictive value (PPV) of a Phase 2 “remember” response for a given reader, assuming exactly one truly known patient in a dataset of size *N* and *N* − 1 non-target patients. The model uses the observed study point estimates for examination-level sensitivity (99/198) and false-positive rate (40/198). **b** Icon-array example of a dataset with 1000 chest radiographs containing one truly known case for that reader. **c** Expected positive “remember” calls in the *N* = 1000 scenario: 0.5 true-positive and 201.8 false-positive calls, yielding a PPV of 0.247%

To apply the study findings to a data-sharing context, we modeled datasets containing one known patient and *N* − 1 non-target patients for a given reader while keeping examination-level sensitivity and false-positive rates constant. Under this low-prevalence model, the positive predictive value (PPV) of a positive “remember” response decreased from 21.6% at *N* = 10 to 9.3% at *N* = 25, 4.8% at *N* = 50, 2.44% at *N* = 100 to 0.247% at *N* = 1000, and 0.0247% at *N* = 10,000 (Fig. 4a). Figure 4b, c shows the example with *N* = 1000: the observed operating characteristics imply 0.5 expected true positives and 201.8 expected false positives, yielding a PPV of 0.247% (rounded to 0.25%). Detailed expected counts across dataset sizes are provided in Appendix E4.

### Exploratory analyses

Exploratory analyses of reader characteristics revealed no significant monotonic relationship between years of radiology experience and performance. Spearman correlation coefficients were 0.22 for sensitivity, 0.14 for specificity, and 0.15 for accuracy. The corresponding coefficients for the time interval between phases were 0.18, −0.02, and 0.09, respectively. These descriptive analyses did not alter the overall interpretation of the study. Detailed correlation coefficients and sex-stratified medians are provided in Appendix E5.

## Discussion

This study was designed as a worst-case stress test of memory-based re-identification. The interval between the first and second exposure was deliberately short, and half of the Phase 2 examinations had been seen before. Despite these favorable conditions for memory, correct recall of at least one explicit identifier occurred in only five of 396 examinations (1.3%). The binary recognition signal was stronger than the explicit identification signal, with 50.0% sensitivity, but it was also noisy, incorrectly judging 20.2% of completely new examinations as remembered.

The distinction between familiarity and identification is important. A sensitivity of 50% may appear concerning in isolation. In this study, however, a positive recognition judgment only rarely translated into a correct identifier. Most positive judgments remained at the level of familiarity without a pseudonym or case-position attempt. Only 3.6% of all positive “remember” responses yielded a correct explicit identifier. From a data protection perspective, the more relevant question is not whether an image feels familiar but whether familiarity can be converted into a correct usable identifier. In the present worst-case scenario, that conversion was rare.

The false-positive rate also deserves attention. One-fifth of the non-target examinations were mislabeled as remembered. This indicates that the recognition signal operated in a noisy regime even under enriched conditions. New images often felt familiar, and this noise diluted the practical meaning of a positive recognition judgment. This becomes especially relevant when prevalence is low. In large de-identified datasets, a reader may know only one patient, or none at all. Under those conditions, even a modest false-positive rate dominates the results, causing most positive recognition judgments to be false matches. More generally, the practical relevance of a positive recognition judgment depends on the ratio between the total dataset size and the number of patients truly known to a given reader. Figure 4c illustrates this: in a dataset of 1000 radiographs with one known case for a given reader, the observed operating characteristics imply 0.5 expected true positives versus 201.8 false positives, yielding a PPV of 0.25%. In other words, the expected number of false-positive “remember” calls is more than 400 times higher than the expected number of true-positive judgments.

The descriptive secondary summary of available non-primary Phase 2 responses points in the same general direction. In those completed readers, positive “remember” judgments were still observed, whereas identifier attempts remained uncommon. Because these data were not fully classifiable at the examination level, they do not replace the predefined primary analysis. They do, however, support the interpretation that noisy familiarity rather than reliable identification was the dominant pattern.

At the same time, empirical risk quantification should not be equated with legal classification. The present study quantifies one specific component of residual privacy risk, namely incidental human memory under enriched recognition conditions. It does not by itself determine whether a dataset would qualify as anonymous in every jurisdiction or deployment context. That assessment may also depend on the availability of linked auxiliary data, access restrictions, organizational safeguards, and the applicable legal standard. In settings in which confirmation through linked local systems is realistically available, a more cautious interpretation may therefore be appropriate.

These data help address a common governance problem in radiology. Ethics committees and data protection officers often need to decide whether a dataset can be reused or shared before empirical information about incidental human-memory risk is available [5, 6]. In such settings, memory-based re-identification is sometimes treated as an undefined or uniformly high risk. The present findings do not suggest that the risk is zero. Some target cases were recognized, and a few identifiers were correctly recalled. However, the findings suggest that the risk is quantitatively limited, even under favorable conditions for memory. For controlled research environments, this argues for proportionate safeguards rather than categorical restrictions. Practical measures, such as access control, audit logging, user agreements, and removal of direct identifiers from images and metadata, remain important [3, 6]. At the same time, the present data do not support treating radiologists’ memory alone as a sufficient reason to block the secondary use of large chest radiograph datasets.

The study also has practical implications for viewer design. When real-world identifiers are displayed in a research or teaching viewer, any recognition event becomes more likely to lead to an identification event. Conversely, when viewers omit direct identifiers, recognition may only result in a vague sense of familiarity. The current findings therefore support the simple design principle of removing direct identifiers by default, especially in shared research environments [3, 8]. However, additional caution may be warranted in settings with higher effective prevalence, such as small local datasets, narrowly defined subspecialty cohorts, or very short temporal intervals between clinical care and research review.

This study had limitations. First, it focused on frontal adult chest radiographs and may not generalize to other imaging settings or examinations with highly distinctive incidental abnormalities [17]. Privacy considerations may also differ in modalities that include recognizable facial anatomy [11, 19]. Second, the minimum 24-h interval and 50% target prevalence were not intended to estimate routine clinical conditions. Interpreting them as real-world prevalence would overstate the opportunity for recognition. In the present study, however, these were features of the upper-bound design. Longer retention intervals, which are common in routine clinical and research workflows, would likely further degrade memory performance [16]. Third, only 33 of 76 readers with complete paired sessions contributed a complete Phase 2 dataset to the predefined primary analysis. This complete-response analysis may have selected more attentive or motivated readers, which could have biased the estimates upward. Fourth, because no post-task questionnaire was administered, we cannot determine whether the frequent absence of identifier attempts reflected retrieval failure, limited confidence, reduced motivation, or a combination of these factors. At the same time, the follow-up items themselves were explicit, and most positive recognition judgments were accompanied by explicit negative follow-up responses rather than by silent missing fields. Fifth, the pool of unique patients was limited. Sixth, the study addressed incidental human memory rather than adversarial attacks, linkage with external data sources, or privacy risks related to data leakage outside of secure environments [4, 5]. Finally, the platform used fictitious pseudonyms rather than real patient names, thereby standardizing explicit recall without revealing actual identities.

In conclusion, even under conditions designed to promote memory retention, radiologists rarely converted familiarity with a prior chest radiograph into accurate recall of an explicit identifier. The main privacy risk signal in this study was not strong identification but rather noisy familiarity, accompanied by frequent false-positive judgments. These findings support treating radiologists’ memory as a limited upper-bound component of re-identification risk and argue for proportionate governance in the secondary sharing of radiographs.

## Supplementary information

ELECTRONIC SUPPLEMENTARY MATERIAL

## Data Availability

The radiographs used in this study were sampled from the publicly available CheXpert dataset. The raw study data (radiologists’ recognition responses) are available from the corresponding author on reasonable request, subject to the data-sharing policies of the German Radiological Cooperative Network (RACOON).

## Abbreviations

CI: Confidence interval
DICOM: Digital Imaging and Communications in Medicine
FN: False negative
FP: False positive
IQR: Interquartile range
NUM: German National Research Network of University Medicine
PPV: Positive predictive value
RACOON: German Radiological Cooperative Network
TN: True negative
TP: True positive
