# Supplementary material for: Radiologists’ memory as a data protection risk: a worst-case stress test for chest radiograph re-identification
Source: Insights Imaging. 2026 Jun 16;17:162. doi: 10.1186/s13244-026-02336-y (PMC13272688; doi:10.1186/s13244-026-02336-y)
Supplement: Supplementary file 1 — ELECTRONIC SUPPLEMENTARY MATERIAL [file 13244_2026_2336_MOESM1_ESM.pdf]

Radiologists' memory as a data protection risk: a worst-case stress test for chest radiograph re-identification
ELECTRONIC SUPPLEMENTARY MATERIAL

Appendix E1: Descriptive summary of available Phase 2 responses outside the predefined primary analysis

Parameter	Primary analysis cohort	Completed readers not included in primary analysis
Readers, *n*	33	43
Available Phase 2 examinations, *n*	396	516
Positive “remember” judgments	139/396 (35.1)	127/516 (24.6)
Any identifier attempt	23/396 (5.8)	17/516 (3.3)
Any identifier attempt among positive “remember” judgments	23/139 (16.5)	17/127 (13.4)
At least one correct explicit identifier	5/396 (1.3)	–

The secondary cohort includes completed readers with available Phase 2 responses who did not contribute a fully classifiable examination-level dataset for the predefined primary performance analysis. Sensitivity, specificity, accuracy, and correctness-based outcomes were therefore not estimated for this group; accordingly, correct explicit identifier recall is not reported for this cohort.

Appendix E2: Random-guessing baseline

Parameter	Observed (%)	Random-guessing baseline (%)	*P*-value vs baseline
Sensitivity	50.0	35.1	< 0.001
Specificity	79.8	64.9	< 0.001
Accuracy	64.9	50.0	< 0.001

Observed examination-level sensitivity, specificity, and accuracy are compared with a random-guessing baseline derived from the overall frequency of “remember” responses (p_yes = 35.1%). Baseline values correspond to expected sensitivity (p_yes), specificity (1 − p_yes), and accuracy under this model. *P*-values are from binomial tests comparing observed numbers of correct decisions with those expected under the baseline.

Appendix E3: Explicit negative follow-up responses after positive recognition judgments in the primary analysis cohort

Parameter	All positive recognition judgments (*n* = 139)	Target positive recognition judgments (*n* = 99)	Non-target positive recognition judgments (*n* = 40)
Pseudonym explicitly not remembered	135 (97.1)	96 (97)	39 (97.5)
Case position explicitly not remembered	120 (86.3)	81 (81.8)	39 (97.5)

Percentages use positive recognition judgments as the denominator. These data summarize the explicit negative follow-up responses recorded after a positive recognition judgment.

Appendix E4: Extrapolated positive predictive value for different dataset sizes

Dataset size (N)	Expected true-positive “remember” calls	Expected false-positive “remember” calls	Expected “remember” calls	PPV among “remember” (%)
10	0.5	1.8	2.3	21.6
25	0.5	4.8	5.3	9.3
50	0.5	9.9	10.4	4.8
100	0.5	20	20.5	2.44
1,000	0.5	201.8	202.3	0.25
10,000	0.5	2,020	2,020.5	0.02
100,000	0.5	20,201.8	20,202.3	≈ 0.00

Hypothetical scenario illustrating the impact of dataset size on memory-based re-identification. Each dataset is assumed to contain exactly one true target patient and N − 1 non-target patients. Expected counts were derived from the observed examination-level sensitivity (99/198) and false-positive rate (40/198). PPV decreases rapidly as N increases, despite the presence of the true patient. More generally, under the observed operating characteristics, PPV falls below 5% once total dataset size is approximately 48 times the number of patients truly known to a given reader and below 1% once it is approximately 246 times that number.

Appendix E5: Exploratory associations between reader characteristics and re-identification accuracy

Parameter	Spearman ρ vs. experience (years)	Spearman ρ vs. time between phases (hours)	Median by sex (male / female)
Sensitivity	0.22	0.18	0.50 / 0.75
Specificity	0.14	-0.02	0.83 / 0.83
Accuracy	0.15	0.09	0.58 / 0.71

Spearman correlation coefficients (ρ) were calculated between each performance metric and reader experience as well as the time interval between phases. Positive values indicate that higher experience or longer time intervals are associated with higher performance. Median performance by sex is reported for readers who reported male or female sex.
